# Tricuspid regurgitation in ischemic mitral regurgitation patients: prevalence, predictors for outcome and long-term follow-up

**DOI:** 10.1186/s12872-021-01982-y

**Published:** 2021-04-21

**Authors:** Ofir Koren, Henda Darawsha, Ehud Rozner, Daniel Benhamou, Yoav Turgeman

**Affiliations:** 1 Heart Institute, Emek Medical Center, Afula, Israel; 2 Internal Medicine D, Emek Medical Center, Afula, Israel; 3 Bruce Rappaport Faculty of Medicine, Technion Israel Institute of Technology, Haifa, Israel; 4 Los-Angeles, CA USA

**Keywords:** Mitral regurgitation, Ischemic Mitral regurgitation, Tricuspid regurgitation, Echocardiography, Heart failure

## Abstract

**Background:**

Functional tricuspid regurgitation (FTR) is common in left-sided heart pathology involving the mitral valve. The incidence, clinical impact, risk factors, and natural history of FTR in the setting of ischemic mitral regurgitation (IMR) are less known.

**Method:**

We conducted a cohort study based on data collected from January 2012 to December 2014. Patients diagnosed with IMR were eligible for the study. The median follow-up was 5 years. The primary outcome is defined as FTR developing at any stage.

**Results:**

Among the 134 IMR patients eligible for the study, FTR was detected in 29.9% (N = 40, 20.0% mild, 62.5% moderate, and 17.5% severe). In the FTR group, the average age was 60.7 ± 9.2 years (25% females), the mean LV ejection fraction (LVEF) was 37.3 ± 6.45 [%], LA area 46.4 ± 8.06 (mm^2^), LV internal diastolic diameter (LVIDD) 59.6 ± 3.94 (mm), RV fractional area change 22.3 ± 4.36 (%), systolic pulmonary artery pressure (SPAP) 48.4 ± 9.45 (mmHg). Independent variables associated with FTR development were age ≥ 65y [OR 1.2], failed revascularization, LA area ≥ 42.5 (mm^2^) [OR 17.1], LVEF ≤ 24% [OR 32.5], MR of moderate and severe grade [OR 419.4], moderate RV dysfunction [OR 91.6] and pulmonary artery pressure of a moderate or severe grade [OR 33.6]. During follow-up, FTR progressed in 39 (97.5%) patients. Covariates independently associated with FTR progression were lower LVEF, RV dysfunction, and PHT of moderate severity. LA area and LVIDD were at the margin of statistical significance (*p* = 0.06 and *p* = 0.05, respectively).

**Conclusion:**

In our cohort study, FTR development and progression due to IMR was a common finding. Elderly patients with ischemic MR following unsuccessful PCI are at higher risk. FTR development and severity are directly proportional to LV ejection fraction, to the extent of mitral regurgitation, and SPAP. FTR tends to deteriorate in the majority of patients over a mean of 5-y follow-up.

**Supplementary Information:**

The online version contains supplementary material available at 10.1186/s12872-021-01982-y.

## Introduction

Acute myocardial infarction directly affects myocardial viability and functionality and leads to structural remodeling and conduction changes seen immediately after an injury. The remodeling process results in valvular dysfunction and eventually hemodynamic changes. Ischemic mitral regurgitation (IMR) is a complication observed following the remodeling of the injured left ventricle [1–3].

IMR is defined as mitral regurgitation following structural remodeling due to ischemic myocardial injury that is not directly related to valvular or sub-valvular pathologies such as mitral valve prolapse, endocarditis, autoimmune disease, medication, or direct radiation effect. The mechanism of IMR seems to be limited to leaflet motion, mainly due to shortening and limited relaxation of the ischemic papillary muscles. IMR is often classified as type IIIb of the Carpentier classification introduced in 1983, involving a restricted leaflet motion, most commonly the posterior leaflet [4]. There can also be a widening of the annulus diameter of the failing LV and secondary leaflet malcoaptation. Ischemic mitral regurgitation may develop after myocardial infarction in 11–59% of patients [5–7].

Functional tricuspid regurgitation (FTR) is the consequence of various structural and functional changes involving the tricuspid annulus and the right ventricle. It is mostly secondary to left-sided mitral pathology in mitral regurgitation, mitral stenosis, and aortic stenosis [8, 9]. Patients with FTR and IMR have a higher risk of developing heart failure and double the mortality risk [10–16]. The prevalence of TR in the context of MV disease was described in recent studies addressing general left-sided pathology [17–21]. The prevalence and natural history of FTR in the setting of mitral regurgitation following myocardial infarction is less known.

## Methods

### Planning of the study

We conducted an observational cohort study using Emek Medical Center's computerized database, a university-based secondary care center. Information regarding patient characteristics, demographics, and angiographic details were collected and analyzed using the Clalit health service database. Patients enrolled in the study were admitted to our heart institute for acute myocardial infarction (MI) from January 2012 to December 2014. Patients younger than 18 years and patients with primary mitral and tricuspid pathology and secondary pulmonary-related tricuspid regurgitation were excluded. The study population consisted of patients who developed mitral regurgitation following myocardial infarction. Patients were eligible for the study provided they met the inclusion criteria (Additional file 1: Table 1s-supplementary) and had at least one transthoracic echocardiogram (TTE) at the time of hospitalization and at least one TTE during follow-up with no new MIs.

### Echocardiographic analysis

All echocardiograms were performed at the hospitalization time using General Electric VIVID-75s, VIVID 95, Philips CVx, and a portable bedside VIVID-1. The echocardiographic reports were prospectively reviewed independently by senior cardiologists using the Carestream Vue imaging system. In case of disagreement, an additional certified cardiologist was consulted. Echocardiographic images were assessed based on mitral and tricuspid insufficiency using an integrated approach considering valvular morphology, the proximal jet's width, color Doppler flow, jet area, and hepatic and pulmonary flow patterns. LV ejection fraction (LVEF) was visually assessed (eyeball) in multiple acoustic windows and calculated using the biplane Simpson method. Continuous-wave (CW) Doppler over TR peak gradient and the vena cava estimated central pressure using inferior vena cava (IVC) diameter and the respiratory fluctuation were used to estimate the systolic pulmonary artery pressures (SPAP) and the right atrial pressure followed by the categorization of severity grade and chamber size according to the American Society of Echocardiography (ASE) and the European Association of Cardiovascular Imaging (EACVI) guidelines [22, 23].

### Sample size

The planned sample size is based on multiple variables such as IMR and FTR prevalence and a group difference of at least 15% to demonstrate a statistical difference of 80% and alpha of 5% in a two-sided test. We had to include a minimum sample size of 164 patients. Preliminary analysis revealed that one out of five patients would be eligible for the study. Following these analyses, we extended the duration of recruitment for 2 years.

### Ethics

Our institutional Ethics Committee approved the study following the Helsinki Convention. The Emek Medical Center IRB waived informed consent due to the use of anonymous patient data and the retrospective nature of the study (approval No. 0105-17, EMC).

### Statistical analysis

Continuous variables were presented using mean ± standard deviation. Categorical variables were presented using frequencies and percentages. A chi-square test was performed to analyze the association between the study groups and categorical variables. For continuous variables, we used the t-test (alternatively the Wilcoxon two-sample test). All confidence intervals are at the 95% level. TR progression was analyzed using the Mantel–Cox multivariant analysis, R-squared analysis, and One-way ANOVA. Multivariable models and linear regression analysis were used to estimate the predictors of FTR. Cut-off points for continuous variables as LVIDD, LA, sPAP, and LVEF were made using the highest sensitivity and specificity point in the ROC curve. The odds ratio was calculated using descriptive analysis in SPSS and linear regression models. Some variables such as age and gender were analyzed using the Cochrane-Armitage test. Forest plot and subgroup analysis for FTR Differences was done using JMP (SAS) and considered statistically significant at the 2-sided p level of 0.05. The statistical analyses were performed using the software SPSS (IBM) and SAS 9.4 (SAS).

### Results of the study

**Fig. 1:**
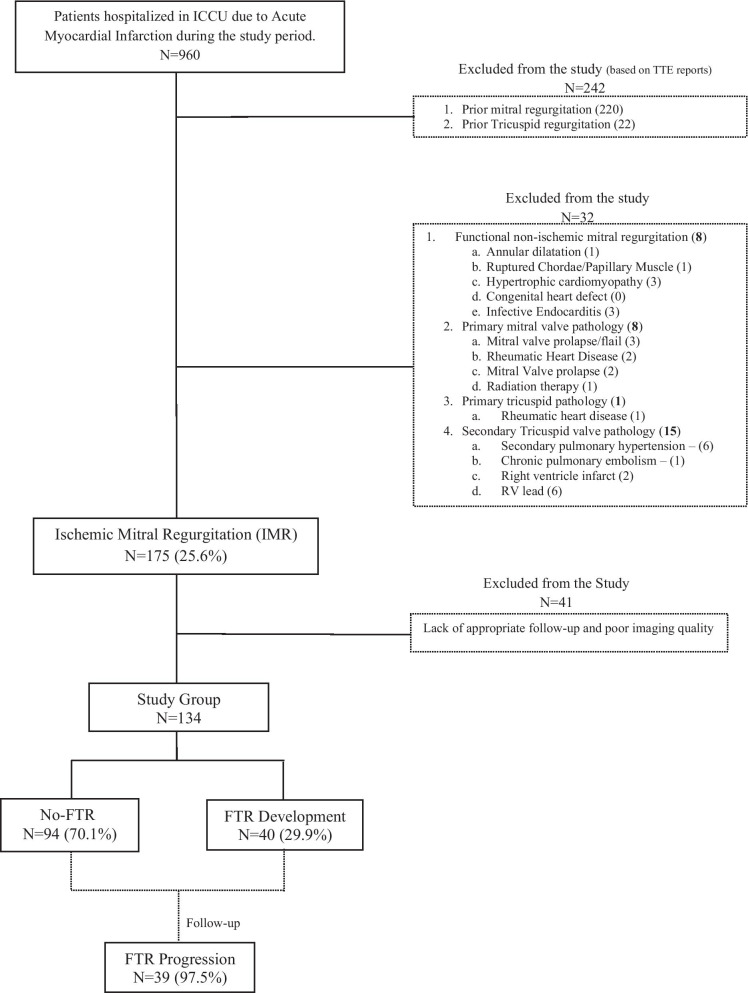
Study design

During the study period, 960 patients were admitted to the intensive cardiac unit due to acute MI. Of these, 274 patients were excluded from the study: 236 patients were excluded due to previous known MR, non-ischemic originating mitral regurgitation, and primary mitral pathology. Thirty-eight patients were excluded due to previous TR, secondary TR, and primary tricuspid pathology. Of the 686 eligible patients, 175 (25.6%) developed ischemic mitral regurgitation. Forty-one patients were excluded due to a lack of adequate follow-up TTE exams and low imaging quality. Thus, 134 patients were in the final study cohort (Fig. 1). The mean follow-up time was 5.2 ± 1.4 years [4.54, 3.54–6.4].

#### FTR development

In the indexed events, FTR was observed in forty patients (29.9%). The two groups' demographic characteristics revealed that patients who developed FTR were older (62.4 ± 11.5 and 59.3 ± 12.4 years, respectively, *p* < 0.0001). Older age was directly linked to FTR severity (59.3 ± 12.4, 55.3 ± 11.9 and 65.4 ± 5.2 years, *p* < 0.0001 using Cochrane-Armitage test for trend, for mild, moderate, and severe FTR, respectively). Multivariate linear regression analysis indicated that failed revascularization during the indexed event was an independent covariate for FTR development and severity (2.1% vs. 25.0% and 28.6%, *p* < 0.0001, R^2^ = 0.134, for failed PCI in Non-FTR patients, FTR patients, and severe FTR patients, respectively). Door-to-balloon (D2B) time of 90 min was achieved in 94% of cases and did not influence FTR development (*p* = 0.56). Gender, smoking, obesity, hypertension, hyperlipidemia, diabetes mellitus, peripheral vascular disease, atrial fibrillation, chronic renal failure, prior coronary bypass surgery, or the Killip class were not found to be different between patients who developed FTR and those who did not (Table 1).

**Table 1:** Baseline characteristics of the study population, overall and by FTR development grade

	Overall (N = 134)	RTF (N = 40)	No FTR (N = 94)	Mild FTR (N = 8)	Moderate FTR (N = 25)	Severe FTR (N = 7)	*p* value for trend
Age, y	59.7 ± 11.5 [59;30–94]	62.4 ± 11.5 [62;37–84]	59.3 ± 12.4 [59;30–94]	55.3 ± 11.9 [55;37–78]	61.0 ± 8.6 [61; 48–84]	65.4 ± 5.2 [66;55–72]	< 0.0001^X^
Age, > 65y, n (%)	43 (32.1)	14 (35)	(30.9) 29	1 (12.5)	8 (32)	5 (71.4)	0.39
Male, n (%)	105 (78.4)	30 (75)	75 (71.4)	7 (87.5)	18 (72)	5 (71.4)	0.23
*Clinical history*							
Smoker, n (%)	75 (56)	20 (50)	51 (54.3)	4 (50)	14 (56)	6 (85.7)	0.5
Obesity, n (%)	(25.4) 34	12 (30)	(23.4) 22	(0) 0	(36)9	(42.9)3	0.42
Hypertension, n (%)	68 (50.7)	19 (47.5)	28 (29.8)	0 (0)	4 (16)	2 (28.6)	0.1
Dyslipidemia, n (%)	95 (70.9)	33 (82.5)	66 (70.2)	6 (75)	19 (76)	4 (57.1)	0.79
Chronic renal failure, n (%)	6 (4.6)	2 (5.0)	5 (5.5)	0 (0)	1 (4)	0 (0)	0.88
Diabetes mellitus, n (%)	52 (38.8)	18 (45)	38 (40.4)	3 (37.5)	7 (28)	4 (57.1)	0.5
PVD, n (%)	7 (5.2)	4 (10)	5 (5.3)	0 (0)	1 (4)	1 (14.3)	0.72
AF, n (%)	26 (19.4)	12 (30)	18 (19)	2 (25)	6 (24)	0 (0)	0.76
CABG, n (%)	6 (4.5)	2 (5.0)	3 (3.2)	1 (12.5)	1 (4)	1 (14.3)	0.28
*Killip class, n (%)*							0.81
1	112 (83.6)	34 (85)	79 (84)	7 (87.5)	19 (76)	7 (100)	
2	1 (0.7)	1 (2.5)	1 (1.1)	0 (0)	0 (0)	0 (0)	
3	7 (5.2)	3 (7.5)	3 (3.2)	0 (0)	4 (16)	0 (0)	
4	14 (10.4)	2 (5.0)	11 (11.7)	2 (8)	2 (8)	0 (0)	
Peak total CPK, mean	1925	1825	1587	1927	2688	3815	0.68
Peak TnI, mean	2713	2302	2531	2612	3091	3927	0.01
*IRA*							0.79
LM	0 (0)	0 (0)	0 (0)	0 (0)	0 (0)	0 (0)	
LAD	64 (47.8)	19 (47.5)	45 (47.9)	4 (50)	10 (40)	5 (71.4)	
CX	4 (3)	2 (5.0)	2 (2.1)	1 (12.5)	1 (40)	0 (0)	
RCA	45 (36) %	14 (35)	31 (33)	2 (25)	11 (44)	1 (14.3)	
Other	21 (15.7)	5 (12.5)	16 (17)	1 (12.5)	3 (12)	1 (14.3)	
Multi-vessels CAD, n (%)	79 (59)	26 (65)	53 (56.4)	6 (75)	16 (64)	4 (57.1)	0.71
Successful PCI, n (%) Ψ	128 (91)	30 (75)	92 (97.9)	8 (100)	17 (68)	5 (71.4)	< 0.0001

PVD indicates Peripheral Vascular disease; AF, Atrial Fibrillation; CABG, Coronary artery bypass grafting; CPK, Creatinine phosphokinase; TnI, Troponin I; IRA, Infarct related artery; LM, left main; LAD, left descending artery; CX, Circumflex artery; RCA, Right coronary artery; CAD, coronary artery disease; PCI, percutaneous coronary intervention

^Ψ^Defined as successfully performed balloon dilatation or stent implantation

^X^Cochrane-Armitage progression test

We did not find a significant statistical correlation between the infarct-related arteries involved or the existence of multiple coronary artery diseases with the development of FTR (*p* = 0.65 and *p* = 0.48, respectively).

**Fig. 2:**
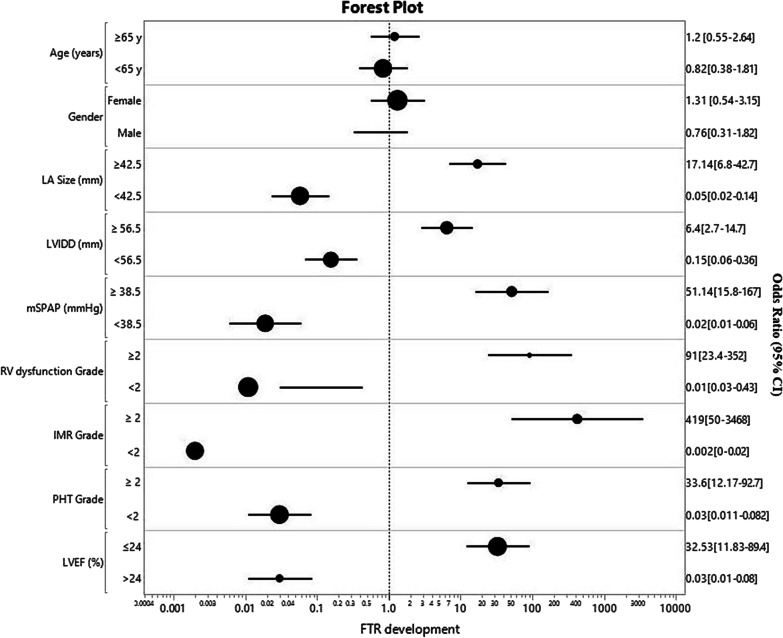
The odds ratio for FTR development in IMR patients [Subgroup Analysis, Log scale]. *p* < 0.0001 [95% CI] for LA size ≥ 42.5, LVIDD ≥ 56.5, mSPAP ≥ 38.5, RV dysfunction grade ≥ 2. IMR grade ≥ 2, PHT grade ≥ 2, LVEF ≤ 24. LVIDD indicates left ventricular internal diameter at end-diastolic phase; LVEF left ventricular ejection fraction; RV FAC, Right ventricular fractional area change; sPAP, systolic pulmonary artery pressure; PHT, Pulmonary Hypertension; MR, mitral regurgitation

Echocardiographic characteristics of the study population showed a significant statistical correlation between LA size, LV internal diastolic diameter (LVIDD), LV ejection fraction (LVEF), RV function (measured as RV fractional area change, RVFAC), Systolic pulmonary artery pressure (SPAP) and MR severity grade with the development of FTR (all *p* < 0.0001) (Table 2). We intended to find a specific cut-off point with the most significant influence on FTR development; therefore, we initially identified all significant predictors that influence FTR development using regression analysis and then applied them on a receiver operating characteristic (ROC) curve. We identified the point with the highest sensitivity and specificity and the area under the curve (AUC) and reanalyzed them in a Cox-regression survival model for Odds-ratio. The following cut-off points and the corresponding odds ratio were identified. LA size ≥ 42.5 mm, LVIDD ≥ 56.5 mm, mSPAP ≥ 38.5 mmHg, and LVEF ≤ 24.0% were best predictors for FTR development (OR 17.1, OR 6.4, OR 51.1, OR 91.6, OR 32.5, respectively). Moderate/Severe MR had the highest odds ratio for FTR development (OR 419.4, 95% CI [50.4–3468.6]) followed by RV with moderate or severe dysfunction (OR 51.4, [95% CI, 15.8–9.6] (Fig. 2).

**Table 2:** Echocardiographic characteristics of study population at the indexed event

	Overall (N = 134)	RTF (N = 40)	No FTR (N = 94)	Mild FTR (N = 8)	Moderate FTR (N = 25)	Severe FTR (N = 7)	p value for Trend	95% CI range
LA Area, mm	38 ± 8.8	46.3 ± 8.06	35 ± 6.89	45.13 ± 8.14	46 ± 8.73	48 ± 5.5	< 0.0001	[− 0.01–0.02]
LVIDD, mm	55 ± 6.2	59.2 ± 3.9	53 ± 6.1	59 ± 1.9	58 ± 4.7	60 ± 1.8	< 0.0001	[0.00–0.03]
LVEF, %	46 ± 9.4	37 ± 6.4	50 ± 7.3	43 ± 3.2	37 ± 5.2	28 ± 2.7	< 0.0001	[− 0.02–0.00]
RV FAC, %	28 ± 5.4	22.2 ± 4.3	30 ± 3.6	23 ± 5.0	21 ± 4.4	22 ± 3.8	< 0.0001	[− 0.06–0.01]
RV dysfunction > 2, n (%)	33 (24)	30 (75)	3 (3.2)	5 (62.5)	20 (80)	5 (71.4)	< 0.0001	[0.46–0.06]
Mean sPAP, mm Hg	36 ± 10.8	48.2 ± 9.4	32 ± 7.1	34 ± 6.4	46 ± 4.6	59 ± 5.3	< 0.0001	[0.01–0.03]
PHT ≥ Moderate, n (%)	33 (24.6)	32 (80)	3 (3.2)	5 (62.5)	20 (80)	5 (71.4)	< 0.0001	[0.0–0.62]
MR > 2, n (%)	47 (35.1)	39 (97.5)	8 (8.5)	7 (87.5)	25 (100)	7 (100)	< 0.0001	[− 0.3–0.39]

Overall and by FTR development grade

R = .395, R2 = .874. The Highest Beta coefficient was observed in LVEF (.396) and RV dysfunction > 2 (.206). No multicollinearity was observed

LVIDD indicates left ventricular internal diameter at end-diastolic phase; LVEF left ventricular ejection fraction; RV FAC, Right ventricular fractional area change; sPAP, systolic pulmonary artery pressure; PHT, Pulmonary Hypertension; MR, mitral regurgitation

#### Predictors of FTR progression

‘FTR progression’ was defined as a deterioration in TR's severity at follow-up compared to the indexed event. During follow-up, FTR progressed in 39 (97.5%) of newly developed FTR patients.

The mean age was 60.5 ± 12.5 years [38–94], and 17.9% were female. There was no statistically significant difference in essential clinical characteristics between patients who developed FTR and patients who did not. The progression of FTR was strongly and independently associated with elevated pulmonary artery pressure (*p* = 0.001 for moderate and severe PHT), reduced LVEF (*p* < 0.0001), and moderate/severe RV dysfunction (*p* = 0.002). LA area and LVIDD were borderline significant (*p* = 0.06 and *p* = 0.05, respectively). Moderate or severe MR was found to be statistically linked to FTR progression (*p* =  < 0.0001, χ^2^ = 0.06, CI = 95%) (Table 3).

**Table 3:** Clinical and echocardiographic characteristics of study population at follow-up

	Overall (N = 134)	RTF progression (N = 39)	No FTR progression (N = 94)	p value for trend
Age, years; mean ± SD (range)	59.7 ± 11.5 [30–94]	60.5 ± 12.5 [38–94]	59.3 ± 11.2 [30–89]	0.356
Gender (Male)	105 (78.4)	32 (82.1)	73 (76.8)	0.06
LA Area, mm (Range)	39.53 ± 9.2 [20–73]	42.9 ± 10.6 [23–73]	35.61 ± 7.0 [20–53]	0.06
LVIDD, mm	56.2 ± 7.2 [42–72]	58.9 ± 7.3 [43–70]	53.1 ± 6.1 [42–64]	0.05
LVEF, %	45.1 ± 10.2 [20–65]	40. 1 ± 10.3 [20–58]	50.6 ± 7.3 [30–65]	< 0.0001
RV FAC, %	27.0 ± 6.1 [10–38]	23.1 ± 6.9 [10–37]	30.50 ± 3.8 [23–38]	0.002
RV dysfunction > 2, n (%)	40 (29.9)	22 (56.4)	3 (4.2)	< 0.0001
Mean sPAP, mm Hg	38.5 ± 12.3 [19–66]	43.6 ± 11.62 [22–65]	31.6 ± 8.4 [19–59]	< 0.0001
PHT ≥ Moderate, n (%)	51 (38.1)	23 (59)	9 (12.5)	0.001
MR > 2, n (%)	46 (34.3)	36 (78.3)	10 (11.4)	< 0.0001
*Echocardiographic Group*^a^				< 0.0001
Group 1		0 (0)	32 (69.6)	
Group 2		5 (5.7)	4 (8.7)	
Group 3		9 (10.2)	5 (10.9)	
Group 4		74 (84.1)	5 (10.5)	

Overall and by FTR development grade

R = .917, R2 = .842. No multicollinearity was observed

LVIDD indicates left ventricular internal diameter at end-diastolic phase; LVEF left ventricular ejection fraction; RV FAC, Right ventricular fractional area change; sPAP, systolic pulmonary artery pressure; PHT, Pulmonary Hypertension; MR, mitral regurgitation

^a^see Table 4 for reference

**Fig. 3:**
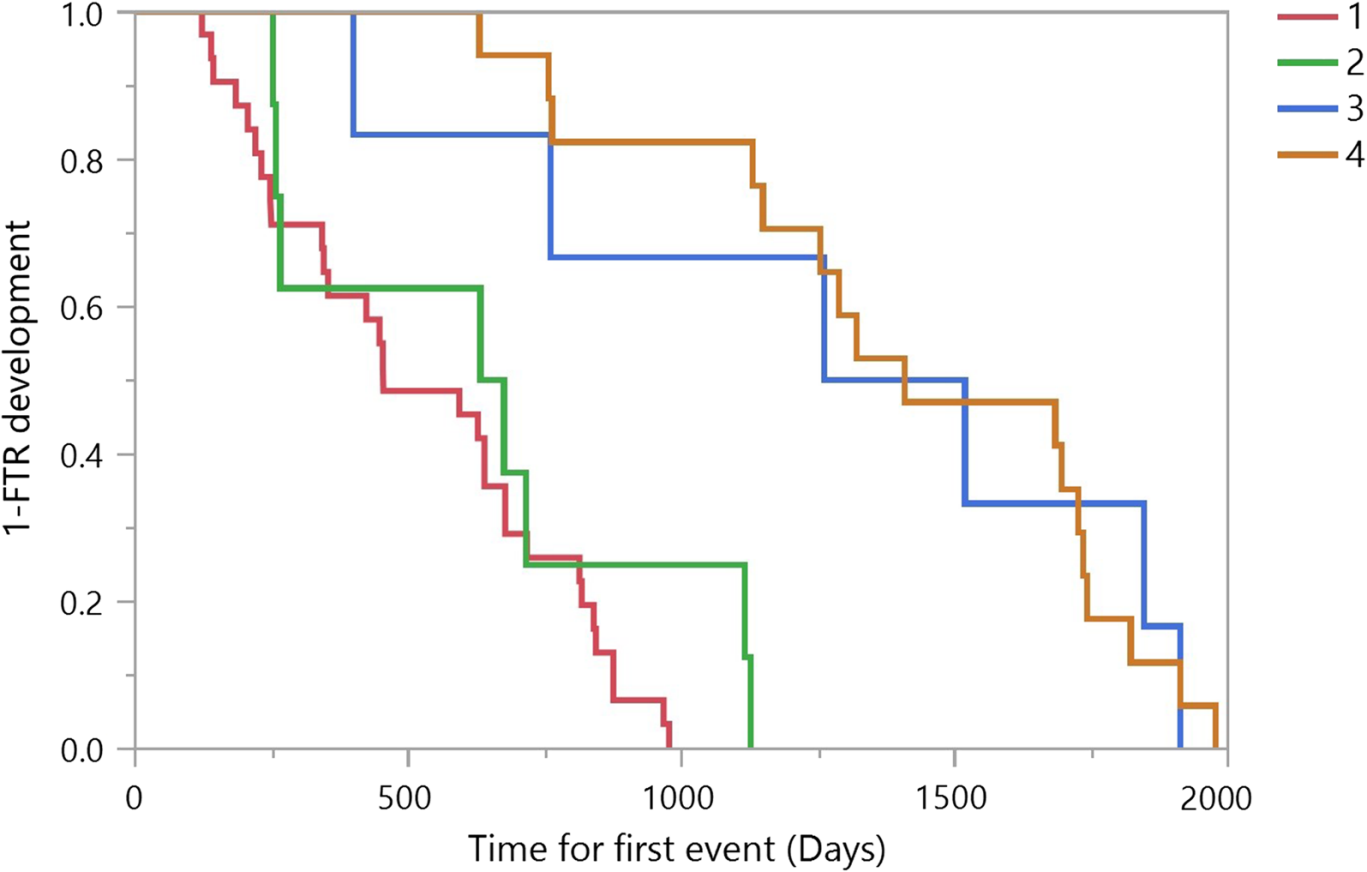
K-M progression curve for FTR development of the study population by four different echocardiographic groups (see Additional file 1: Table S1 for reference). *p* < 0.0001 for the difference between groups (Log-rank and Wilcoxon tests)

The mean time to progression rate was 4.1 ± 1.8 years [3.9, 2.4–5.4]. To assess whether a specific group of patients had a higher progression rate, we created four arbitrary groups based on the most common combination of echocardiographic characteristics in the study population. Group 1 included patients with MR grade ≥ 2, LVEF ≤ 24%, moderate/severe RV dysfunction and SPAP ≥ 38 mmHg, while Group 4 included patients with none of the above criteria (MR grade < 2, LVEF > 24%, normal or mild RV dysfunction and SPAP < 38 mmHg). Group 2 and group 3 had mixed echocardiographic criteria (Table 4). The progression rate was the highest in Group 1 (2.5 ± 1.1 years [2.1, 0.6–5.2] and the lowest in group 4 (4.7 ± 2.1 [5.04, 3.2–5.4] (Fig. 3).

**Table 4:** Echocardiographic parameters according to Groups (reference for Fig. 3)

Echocardiographic parameters	Group 1	Group 2	Group 3	Group 4
	(n = 23)	(n = 9)	(n = 41)	(n = 97)
MR grade ≥ 2	+ ^a^	+	+ ^e^	–
LVEF ≤ 24%	+ ^b^	+	–	–
RVD grade ≥ 2	+	–	–^f^	–^h^
sPAP ≥ 38 mmHg	+ ^c^	+ ^d^	–^g^	–^i^

MR indicates mitral regurgitation; LVEF, Left ventricular ejection fraction; RV, right ventricular dysfunction; sPAP, systolic pulmonary artery pressure

^a^Two patients had MR grade < 2

^b^Seven patients had LVEF > 24%

^c^One patient had sPAP < 38 mmHg

^d^Two patients had sPAP < 38 mmHg

^e^Five patients had MR grade < 2

^f^Six patients had LVEF > 24%

^g^Five patients had sPAP ≥ 38 mmHg

^h^One patient had RVD grade ≥ 2

^i^Ten patients had sPAP ≥ 38 mmHg

**Fig. 4:**
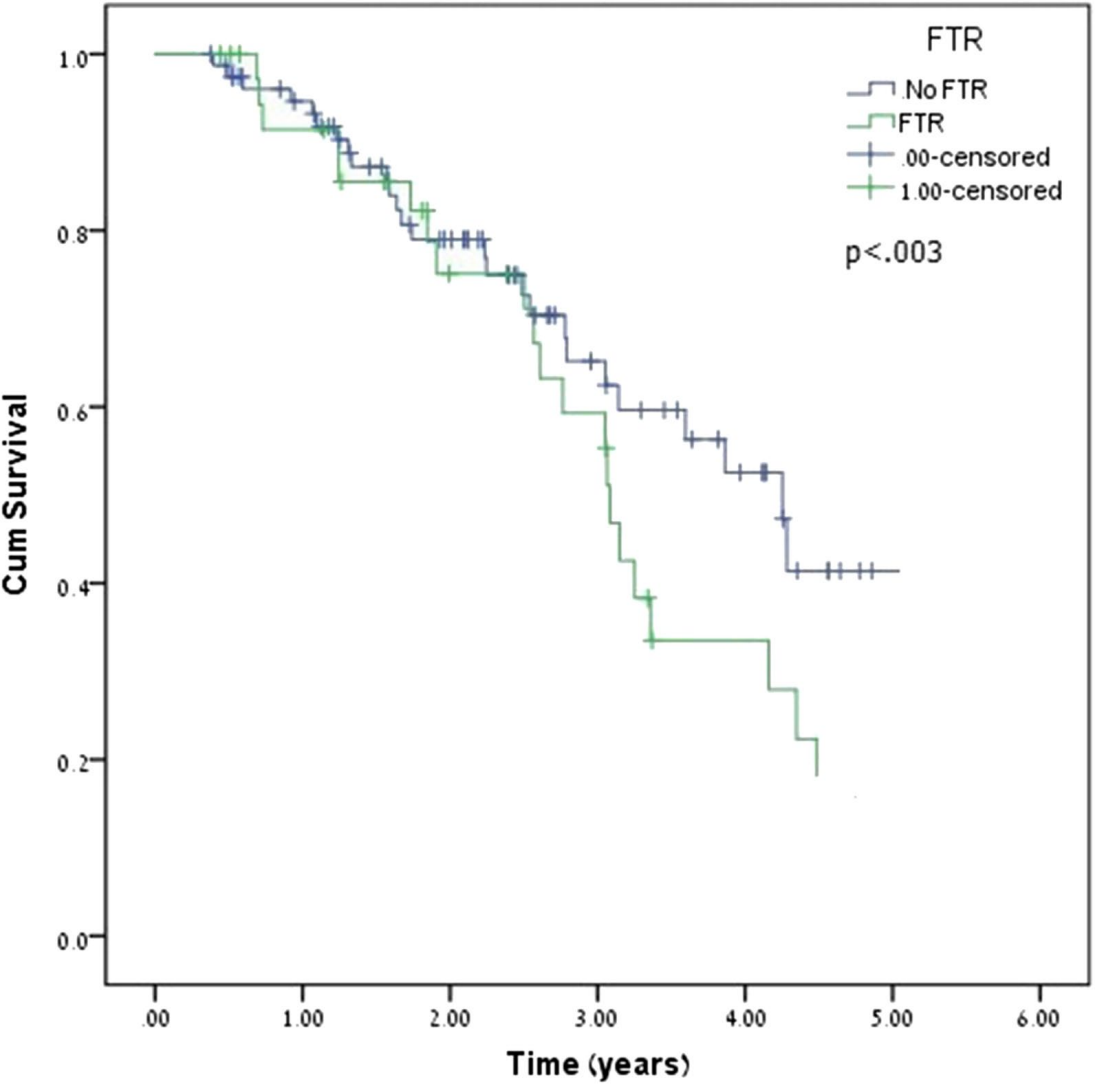
K-M survival curve of FTR development among the study population

The presence of FTR was a significant predictor of increased mortality in patients with IMR (Mantel-Cox χ^2^ = 5.02, *p* = 0.025). The mean survival time was 4.58 years (95% CI: 4.11–5.04 years) in the FTR group and 5.15 years (95% CI: 4.9–5.4 years) in the no-FTR group (Fig. 4).

## Discussion

Our study results support recent reports of bivalvular deterioration in heart failure patients with reduced ejection fraction (HFrEF) and the interconnection between the mitral and tricuspid apparatuses [24–27]. Our study highlights a specific group of patients with ischemic-related mitral regurgitation and its effect on functional TR development.

Multivalve involvement was described in the past regarding rheumatic heart disease and congenital heart disease and not extensively in the context of ischemic heart disease [28–32].

Myocardial infarction is still the most common cause of HFrEF, and until recently, it was thought that its primary effect was directed toward left-sided pathology through an extensive remodeling process [33, 34].

IMR is a common complication after myocardial infarction that requires adequate follow-up regardless of the appearance of clinical symptoms [35–37]. Mild asymptomatic IMR usually tends to be neglected; however, our study demonstrates a linear relationship between mitral regurgitation and the development of functional tricuspid regurgitation due to myocardial infarction. This linear connection involves volume and pressure overload, shifting from the left to the right side through the pulmonary vasculature.

Over a five-year follow-up, we could see that FTR developed in nearly a third of IMR patients, and more surprisingly, it progressed in almost all newly developed FTR patients. Elderly patients who had unsuccessful PCI seemed to be at a higher risk than others. We identify several echocardiographic predictors for FTR development and rapid FTR deterioration and a cut-off threshold for better monitoring and future management.

Our findings are consistent with prior reports of left and right chamber interconnection. Deterioration of left side chambers resulting from extensive remodeling may shift volume and pressure initially toward unadjusted right-sided chambers and result in larger LA size and systolic pulmonary artery pressure. Over time, without proper optimal heart failure medical therapy, this may result in RV dysfunction and progressive development of FTR.

Bivalvular regurgitation, particularly with the tricuspid valve's involvement, dramatically influences the prognosis of heart failure, limits therapeutic options, and substantially increases the overall mortality [38–41].

IMR should be appropriately monitored and followed up regardless of symptoms. FTR should be assessed annually to avoid significant deterioration and start prompt treatment for heart failure before the first signs of heart failure appear.

## Limitation of the study

Our study is designed in a retrospective fashion. The data analysis was retrospectively reviewed on computerized patient medical records. Echocardiographic images were assessed according to the ASE criteria.

Quantification of the severity of mitral and tricuspid regurgitation was based on qualitative grading. These assessment methods may be confounded by several technical and hemodynamic factors, including the jets' eccentricity and atrial size. However, we cannot accurately apply quantitative or semiquantitative methods because of the nature of the study.

The influence of heart failure medical therapy and its contribution to valvular dysfunction could not be evaluated here due to a lack of clinical data. Larger samples are needed to evaluate these factors.

## Conclusion

In our cohort study, FTR was a common finding in IMR patients. Third of patients with ischemic mitral regurgitation developed functional tricuspid regurgitation (FTR). Elderly patients who had unsuccessful PCI are at a higher risk. FTR progression was observed in almost all patients over a 5-years follow-up period. Rapid FTR progression was observed in patients with moderate and severe MR, LVEF ≤ 24%, LA size ≥ 42.5 mm, LVIDD ≥ 56.5 mm, and RV of moderate and severe dysfunction. FTR was found to be positively linked to poor outcomes and lower survival rates.

## Supplementary Information

**Additional file 1.** Supplementary file.

## Data Availability

The datasets used and analyzed during the current study are available from the corresponding author on reasonable request.

## Abbreviations

IVC: Inferior vena cava
LV: Left ventricle
RV: Right ventricle
PAP: Pulmonary arterial pressure
LVEDD: Left ventricular end diastolic diameter
LVESD: Left ventricular end systolic diameter
LVEF: Left ventricular ejection fraction
RVEF: Right ventricular ejection fraction
CWD: Continuous wave Doppler
