# Supplementary material for: Tricuspid regurgitation in ischemic mitral regurgitation patients: prevalence, predictors for outcome and long-term follow-up
Source: BMC Cardiovasc Disord. 2021 Apr 21;21:199. doi: 10.1186/s12872-021-01982-y (PMC8058984; doi:10.1186/s12872-021-01982-y)
Supplement: Supplementary file 1 — Additional file 1. Supplementary file. [file 12872_2021_1982_MOESM1_ESM.docx]

**Tricuspid Regurgitation in Ischemic Mitral Regurgitation Patients - Prevalence, Predictor for Outcome and Long-term Follow-Up**

Ofir Koren, MD^1,3^ Henda Darawsha, MD^2^, Ehud Rozner, MD^1^, Daniel Benhamou, Yoav Turgeman, MD^1,3^

^1^Heart Institute, Emek Medical Center, Afula, Israel

^2^Internal Medicine D, Emek Medical Center, Afula, Israel

^3^Bruce Rappaport Faculty of Medicine, Technion Israel Institute of Technology, Haifa, Israel

Corresponding author:

Ofir Koren, MD, FICA

Ofirko1@clalit.org.il

Tel: 972-46495121

Fax: 972-48141414

Address: Norit 40, Kfar-Tavor Israel 1524100

Supplementary

Tables legends

Table 1s. Inclusion and Exclusion Criteria

Table 1s. Inclusion and Exclusion Criteria

| Inclusion Criteria | Exclusion Criteria |
| --- | --- |
| 1. Age over 18 2. Acute myocardial infarction | 1. Primary Mitral Valvular Pathology - Hypertrophic/dilated cardiomyopathy, rheumatic mitral disease, prosthetic valve. mitral valve prolapse, Iatrogenic mitral regurgitation 2. Primary Tricuspid valve pathology – rheumatic heart disease, Congenital (ebstein anomaly, Tricuspid dysplasia, Tricuspid hypoplasia, Tricuspid cleft, double orifice, unguarded tricuspid valve orifice), Endocarditis, Endomyocardial fibrosis, Carcinoid disease, Traumatic mitral pathology (blunt chest injury, laceration), pace-maker/defibrillator lead interference, right ventricular biopsy, Drugs (fen-phen), chest radiation. 3. Secondary Tricuspid valve pathology d/t structural left sided pathology - Primary pulmonary hypertension, Secondary pulmonary hypertension (d/t chronic lung disease, chronic pulmonary embolism, left-to-right shunt), ARVD^a^, DCMP^b^, Right ventricle Infarct. Atrial fibrillation, Cardiac tumors (particularly right atrial myxomas) 4. Unsatisfactory Echocardiography follow up images or Poor Echogenic windows 5. Pregnancy |

^a^ ARVD - Arrhythmogenic Right Ventricle Dysplasia

^b^ DCMP – Dilated Cardiomyopathy
